# CircMTO1 inhibits ox-LDL-stimulated vascular smooth muscle cell proliferation and migration via regulating the miR-182-5p/RASA1 axis

**DOI:** 10.1186/s10020-021-00330-2

**Published:** 2021-07-08

**Authors:** Ningning Ji, Yu Wang, Xinyan Gong, Shimao Ni, Hui Zhang

**Affiliations:** Department of Cardiology, Yiwu Central Hospital, Affiliated Hospital of Wenzhou Medical University, No.699, Jiangdong Road, Yiwu City, 322000 Zhejiang Province People’s Republic of China

**Keywords:** CircMTO1, miR-182-5p, RASA1, VSMCs, Proliferation, Apoptosis

## Abstract

**Background:**

Circular RNAs (circRNAs) play critical roles in the development of atherosclerosis (AS). This study investigated the role of circMTO1 in the progression of AS.

**Methods:**

Serum samples from AS patients and healthy volunteers and vascular smooth muscle cells (VSMCs) were used as the study materials. The expressions of circMTO1 and miR-182-5p were measured by RT-qPCR. The effects of circMTO1, miR-182-5p, and RASA1 on VSMC proliferation and apoptosis were examined by MTT and BrdU assays and wound healing and flow cytometric analyses, respectively. Downstream target genes of circMTO1 and miR-182-5p were predicted using target gene prediction and screening and confirmed using a luciferase reporter assay. RASA1 expression was detected by RT-qPCR and Western blot.

**Results:**

circMTO1 expression was decreased, while miR-182-5p expression was increased in human AS sera and oxidized low-density lipoprotein (ox-LDL)-stimulated VSMCs. CircMTO1 overexpression inhibited the proliferation and promoted the apoptosis of ox-LDL-stimulated VSMCs. CircMTO1 was found to be served as a sponge of miR-182-5p and RASA1 as a target of miR-182-5p. Moreover, circMTO1 acted as a ceRNA of miR-182-5p to enhance RASA1 expression. Furthermore, miR-182-5p overexpression and RASA1 knockdown reversed the effects of circMTO1 overexpression on the proliferation, migration, and apoptosis of ox-LDL-stimulated VSMCs.

**Conclusion:**

CircMTO1 inhibited the proliferation and promoted the apoptosis of ox-LDL-stimulated VSMCs by regulating miR-182-5p/RASA1 axis. These results suggest that circMTO1 has potential in AS treatment.

**Supplementary Information:**

The online version contains supplementary material available at 10.1186/s10020-021-00330-2.

## Background

Arteriosclerosis (AS) has become one of the major causes of increased mortality and incidence rates of cardiovascular and cerebrovascular diseases worldwide (Lippincott and Wilkins 2017; Tabib et al. 2017). It has seriously affected the quality of life of the middle-aged and the elderly and increased the social burden (Asayama et al. 2017). The leading pathological cause and characteristic of this chronic inflammatory disease is the hardening of the arterial wall and narrowing of the lumen caused by atherosclerotic plaques (Tölle et al. 2015). Lower extremity arteriosclerosis obliterans (ASO) is the most common occlusive disease of peripheral blood vessels, and AS can cause ASO. The pathogenesis of AS has been extensively studied and explored. At present, the mechanism of activation of vascular smooth muscle cells (VSMCs) has gradually attracted scholars’ attention (Zhao et al. 2014; Begum et al. 2011). In the early stages of the disease, the main feature is that abnormal proliferation of VSMCs leads to intimal thickening and lumen shrinkage (Rotllan et al. 2015). It is well known that the proliferation of VSMCs is the common pathogenesis of cardiovascular and cerebrovascular diseases. Due to the limitations in the choice of clinical treatment and the prognosis effect (Zhang et al. 2011), more and more scholars have begun to pay attention to the pathogenesis of arteriosclerosis, hoping to interfere or block the occurrence of arteriosclerosis.

Oxidized low-density lipoprotein (ox-LDL) has been proven to be involved in the formation and progression of atherosclerotic plaque (Li et al. 2017). Studies have shown that a low concentration of ox-LDL can promote the proliferation of VSMCs, while a high concentration of ox-LDL can promote the apoptosis of VSMCs (Wang et al. 2019a). Therefore, in this study, ox-LDL was used to establish the proliferation model of VSMCs to explore the mechanism of atherosclerosis further.

Many basic studies in recent years have confirmed that the expression profiles of many genes are often changed in the pathophysiology of cardiovascular diseases (Wallace et al. 2008). In recent studies, it has been reported that circular RNA (circRNA) is involved in the pathophysiological process of cardiovascular disease, diabetes, and cancer (Gruner et al. 2016; Ashwal-Fluss et al. 2014). More and more basic research results have shown that circRNA plays a critical role in the occurrence and progression of cardiovascular diseases (Fan et al. 2017; Holdt et al. 2018). For example, studies have found that multiple circRNAs act as potential biomarkers in coronary atherosclerosis. A variety of circRNAs in circulating blood are beneficial to the screening and early diagnosis of coronary atherosclerotic heart disease (Qu et al. 2017). CircMTO1 is a type of circRNA which is conserved in mouse and human (Additional file 1). It has been found that circMTO1 is abnormally expressed in many diseases, such as liver cancer and glioblastoma (Han et al. 2017a). However, it is unclear whether circMTO1 is involved in the proliferation of VSMCs.

Numerous studies have shown that circRNAs regulate gene expression by acting as miRNA sponges to competitively bind to messenger RNAs (mRNAs) and proteins to form a complex, regulating mRNA splicing, translation, and degradation (Jin et al. 2016). More than 2000 miRNAs have been discovered in the human genome (Anokyedanso et al. 2011). MiRNAs play essential roles in the development of tumors, endocrine diseases, nervous system diseases, and cardiovascular diseases, to name a few (Chen et al. 2017; Piwecka et al. 2017). Recent studies have found that miRNAs participate in controlling VSMCs’ proliferation. MiR-132, miR-22, miR-379 and miR-124 inhibit, while miR-214, miR-221 and miR-146 promote VSMCs’ proliferation (Guo et al. 2016a; Li et al. 2018). Previous studies have shown that miR-182-5p is involved in cancer, myocardial ischemia and reperfusion-induced injuries, and spinal neuronal diseases (Li et al. 2014; Xue et al. 2016). However, whether miR-182-5p is involved in VSMCs’ proliferation is not fully understood. RASA1 is a cytoplasmic protein with a molecular weight of 120 kD. Its C-terminus is a GTPase-activated protein (GAP) domain with catalytic activity (Gong et al. 2015). Its N-terminus consists of PH, CaLB/C2, SH2, and SH3 domains. RASA1 activates Ras GTPase, which hydrolyzes Ras GTP and inhibits Ras signaling pathway. Studies have shown that RASA1 promotes cell apoptosis by inhibiting the Ras-MAPK pathway, which is closely related to various diseases (Revencu et al. 2013; Hancock et al. 2014). Therefore, we hypothesized that circMTO1 inhibits VSMCs’ proliferation by modulating miR-182-5p/RASA1 axis. The study’s main purpose was to investigate the mechanism by which circMTO1 regulates VSMCs’ proliferation with the hope to provide new ideas for the diagnosis and targeted therapy of AS and a theoretical basis and experimental evidence for elucidating the mechanism of circRNA-related vascular lesions and finding potential therapeutic targets.

## Materials and methods

### Clinical samples

This study was conducted with the approval of the Yiwu Central Hospital’s Ethics Committee, and all participants signed the informed consent (50–70 years old, 40% female). Blood samples were collected from AS patients (n = 55) and healthy volunteers (n = 55) in centrifuge tubes without anticoagulants. Serum was extracted by centrifugation for RNA isolation using TRIzol LS reagent (Yanjin, Shanghai, China). None of the healthy volunteers had AS disease, malignant tumors, and inflammatory diseases (< 1 month).

### Cell culture

Human aortic vascular smooth muscle cells (HA-VSMC) were obtained from the Fenghui Biotechnology Co., Ltd (Hunan, Shanghai, China) and maintained in F-12 K medium supplemented with 10% FBS (Invitrogen) at 37 °C in a humidified atmosphere containing 5% CO_2_.

### Cell transfection and treatment

The full-length of circMTO1 was amplified by PCR and cloned into pcDNA3.1 vector (Invitrogen) to construct a pcDNA-circMTO1 overexpression plasmid. Si-circMTO1 and its control si-NC, si-RASA1 and its control si-NC, and miR-182-5p mimic (miR-182-5p) and its control miR-NC were all designed and synthesized by GenePharma (Suzhou, China). VSMCs were treated with ox-LDL (Luwen, Shanghai, China) to detect the expression of circMTO1 and miR-182-5p. CircMTO1, miR-182-5p, and RASA1 sequences are provided in Additional file 1.

### qRT-PCR

Total RNA in cells was extracted using TRIzol LS reagent (for serum RNA extraction) (Yanjin, Shanghai, China). microRNA was extracted and purified using TaqMan ABC miRNA Purification Kit (Thermo Fisher Scientific, USA). After the reverse transcription reaction, qRT-PCR was performed using a ViiATM 7 real-time PCR system (Life Technologies, Grand Island, NY). The miR-182-5p level was normalized to U6, and circMTO1, MTO1 and RASA1 were normalized to GAPDH. The relative expression levels of each gene were calculated and normalized using the 2^−ΔΔCt^ method. The specific qRT-PCR for circMTO1, MTO1, and GAPDH were performed as previously reported (Li et al. 2013) with the following primers: CircMTO1 (divergent primer): forward 5′-GGCCATCCTATGTCAGTTG-3′; CircMTO1 (divergent primer) reverse 5′-AGGTAGGCCCGCACGGT-3′; MTO1 (convergent primer) forward: 5′-AAGTG CCGTTGGGTGTGG-3′; MTO1 (convergent primer) reverse: 5′-CAATCATTCGTT GGAGGTT-3′; MiR-182-5p forward: 5′-CCCAACT GTATGGTTT-3′; MiR-182-5p reverse: 5′-CGGATGGCCCAACGG-3′; RASA1 forward: 5′-TCGAAAAGCTATGC TATGGC-3′; RASA1 reverse: 5′-CTAACAATCACGTGCGCGA-3′; GAPDH forward: 5′-CGAGAGAGCGATCAGACCT-3′; GAPDH reverse: 5′-GTATAGTTGCT CACGGGAAC-3′; U6 forward: 5′-ATGTGGTATGACACCTGGGCC-3′ and U6 reverse: 5′-GATTGGCAGCGATTATACACC-3′.

### Cell proliferation assay

For the CCK-8 assay, transfected cells were seeded into 96-well plates at a density of 2000 cells per well. After 48 h, cell viability was measured by the Cell Counting Kit-8 (CCK-8) system (Liji, Shanghai, China). The absorbance at 450 nm of each well was measured using a microplate reader (Tecan, Switzerland). For the BrdU incorporation assays, transfected cells were seeded in 96-well plates at a density of 2000 cells per well. After 48 h, cell proliferation was analyzed using the BrdU Cell Proliferation Assay Kit (#5213S, Cell Signaling).

### Cell apoptosis assay

The transfected cells were collected, washed, and incubated with 500 μl of binding buffer, 5 μl of FITC Annexin V, and 5 μl of propidium iodide (PI). The apoptotic rate was determined using flow cytometry (FACS Calibur, USA) following the manufacturer’s instructions.

### Dual-luciferase reporter gene assay

The wild-type or mutant sequences of the circMTO1 or RASA1 3′-untranslated region (3′-UTR) were cloned into the pmirGLO reporter plasmids. VSMCs were co-transfected with pcDNA3.1, pcDNA-circMTO1 (wild type or mutant), the negative control, or miR-182-5p. After 48 h of transfection, luciferase activity was measured using a dual-luciferase reporter assay system (Promega).

### Wound healing assay

24 h before transfection, cells were digested and passaged to 12-well plates. The cell confluency at transfection was 60–80%. The experimental group and the control group were set up parallelly. The transfection concentration was 50 nmol/L. 24 h after transfection, a tip was used to draw a cell-free area of substantially the same width in each well to mimic wounds. Capecitabine (Abcam, USA), as the DNA replication inhibitor, was added into the culture media at a final concentration of 12 nM. The cells were then cultured for 24 h to observe their ability to migrate.

### Western blot

The transfected cells were collected, and total proteins were extracted and quantified using the BCA Protein Assay Kit. After separated and transferred onto PVDF membranes, proteins were incubated with antibodies against RASA1 (1:500; Abcam, ab40677), a-SMA (1:500; Abcam, ab5694), LDL-receptor (1:1000; Abcam, ab52818), caspase 3 (1:1000; Abcam, ab32315), cyclin D1 (1:1000; Abcam, ab16663) and GAPDH (1:1000; Sangon, Shanghai), overnight at 4 °C respectively. After washed, the proteins were incubated with 1:2000 HRP-labeled anti-rabbit secondary antibody (Catalogue No. ab150077) for 1 h and visualized using ECL reagent. Western blot analyses were performed as previously reported (Kuhar and Yoho 2015).

### AS animal model

A total of 15 10-week-old male ApoE ^−/−^ C57BL/6 mice from the Animal Center of Shanghai (Shanghai Lab.Animal Research Center) were used in the study. These mice were fed on a Western-type diet (contain 21% fat by weight, 0.15% cholesterol) for 5 weeks to generate atherosclerotic plaques (Wang et al. 2019b), AAV-pcDNA, AAV-pcDNA-circMTO1, and AAV-pcDNA-circMTO1/AAV-miR-182-5p were injected into these mice via tail veins (1 × 10^11^ PFU/ml, 1 μL) once every two weeks, respectively, mice were then fed on a chow diet for the next 30 weeks, and sacrificed. Aortas from the base at the aortic valve up to the diaphragm were collected as described previously (Paigen et al. 1987) (Table 1).

**Table 1:** Antibodies list

Name	Reactivity species	Application	Company	Cat. .No
RASA antibody	Mouse, human	WB	Abcam	Ab40677
α-SMA antibody	Mouse, human	IHC, WB	Abcam	Ab7817
LDL-receptor antibody	Mouse, human	WB	Abcam	Ab30532
Caspase3 antibody	Mouse, human	WB	Abcam	Ab13847
Cyclin D1 antibody	Mouse, human	WB	Abcam	Ab16663

### Histological evaluation

After the AS model was established, the whole aorta from the heart to the abdominal aorta was dissected. Atherosclerotic plaques in the aortas were stained with 0.5% oil-red O (Sigma-Aldrich) for 15 min at room temperature. Serial cryosections (8 μm) were cut along the aortic root specimens at − 20 °C using a cryotome (HM550, Thermo Scientific, USA). The sections were tained with hematoxylin/eosin and oil-red O and counterstained with DAPI to evaluate the lipid content in the plaque areas.

### Immunochemistry

OCT-embedded myocardial tissue sections were used for immunochemistry. Serial sections of the aortic root beyond the end of the aortic sinus were selected for staining. Rabbit obtained antibodies for α-actin (Abcam, 0.5 μg/ml) was chosen for probing the smooth muscle and foam cells. Goat anti-rabbit antibodies were also used as secondary antibodies. The staining images were captured using a Leica microscope.

### Statistical method

The monitoring data were analyzed by SPSS19.0 statistical software. Results of the data analysis were shown as mean ± standard deviation (mean ± SD). Multigroup data analysis was based on one-way ANOVA followed by the LSD test. P < 0.05 indicated a significant difference.

## Results

### CircMTO1 expression was reduced in the serum of AS patients and ox-LDL-stimulated VSMCs

**Fig. 1:**
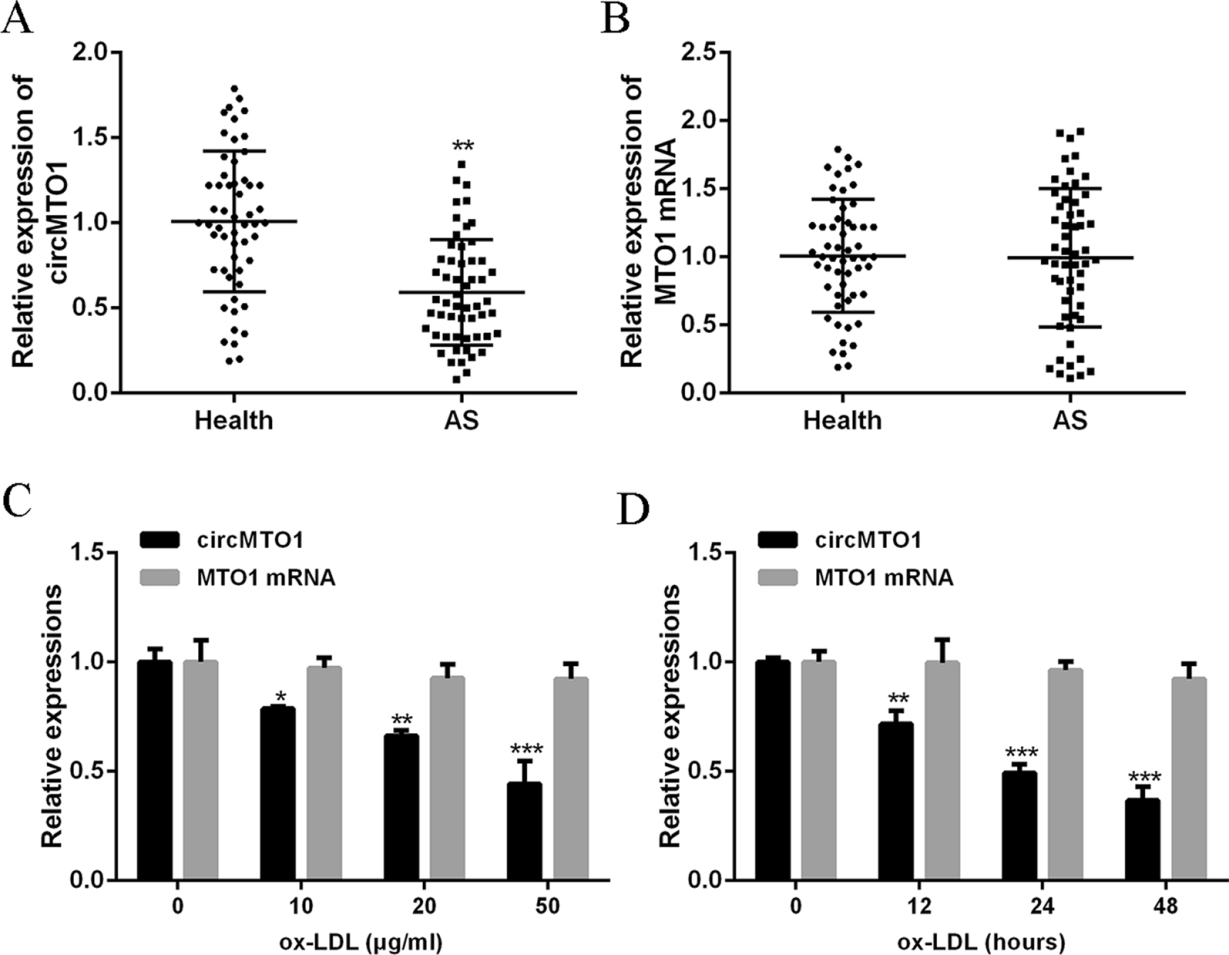
Reduced circMTO1 expression in serum and ox-LDL-stimulated VSMCs. **A**, **B** The mRNA levels of circMTO1 and MTO1 in serum of AS patients (n = 55) and healthy volunteers (n = 55). **C** The mRNA levels of circMTO1 and MTO1 in VSMC treated with ox-LDL (0, 10, 20, 50 μg/ml) for 24 h. **D** The mRNA expression level of circMTO1 and MTO1 after treated with ox-LDL for different times (0, 12, 24 and 48 h).*P < 0.05, **P < 0.01 and ***P < 0.001

Firstly, circMTO1 expression in serum of AS patients and ox-LDL-stimulated VSMCs was examined. As shown in Fig. 1A, circMTO1 expression was significantly lower in AS patients (n = 55) than that in the health group (n = 55) (P < 0.01). Moreover, there was no significant difference in MTO1 mRNA expression (P > 0.05) (Fig. 1B). As shown in Fig. 1C and D, with ox-LDL concentration increasing and action time prolonging, circMTO1 expression in VSMCs gradually decreased (P < 0.01), while no significant difference was found in MTO1 mRNA expression. Based on the results, 50 μg/ml ox-LDL treatment for 48 h was selected for subsequent experiments. These results demonstrate that circMTO1 plays a role in the progression of AS.

### CircMTO1 overexpression inhibited proliferation and migration of ox-LDL-stimulated VSMCs

**Fig. 2:**
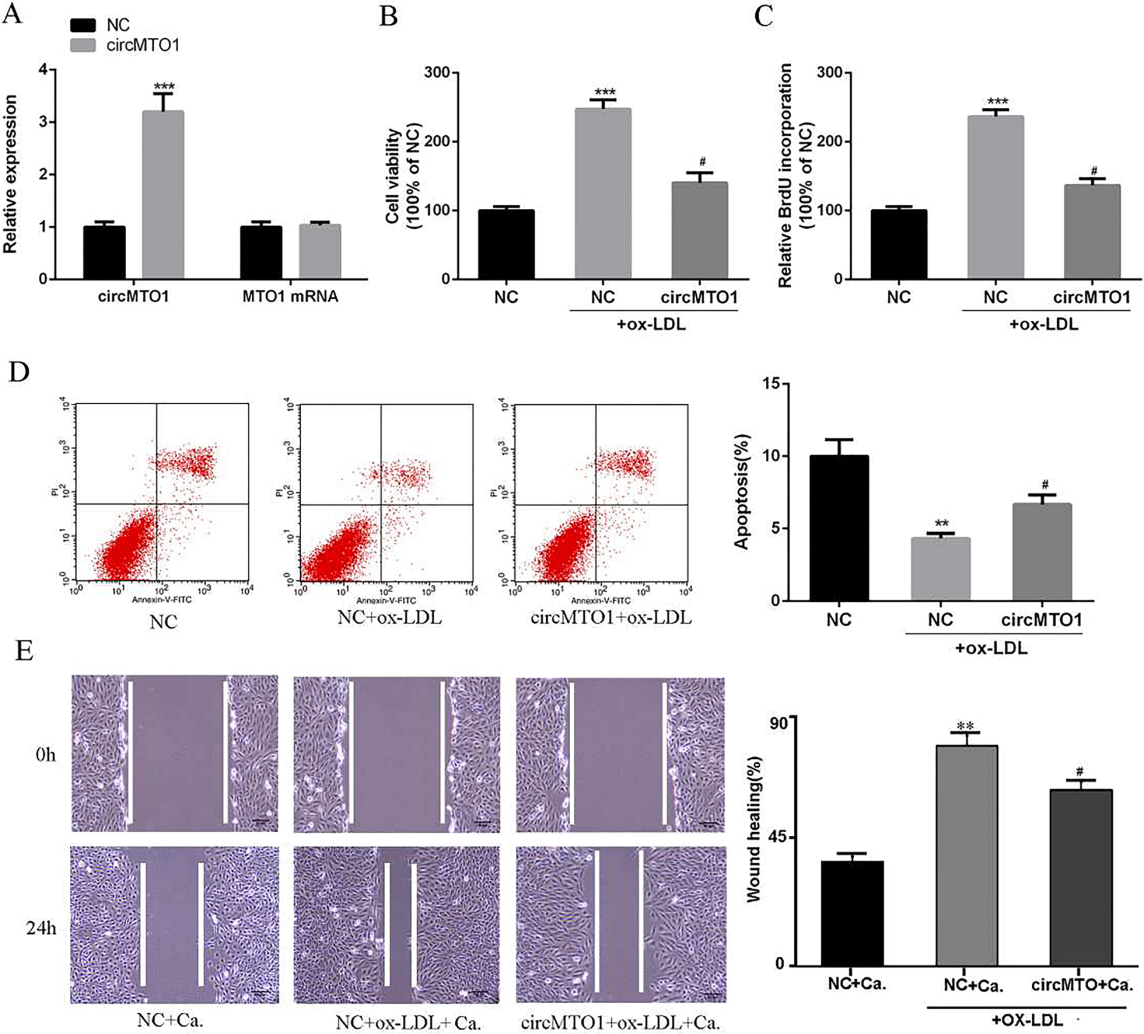
CircMTO1 inhibited the proliferation and migration and induced the apoptosis of ox-LDL-stimulated VSMCs. **A** The mRNA expression levels of circMTO1 and MTO1 in VSMCs transfected with pcDNA 3.1-circMTO1. **B**, **C** The effect of circMTO1 overexpression on cell viability and proliferation determined by CCK8 and BrdU incorporation assays. **D** The effect of circMTO1 overexpression on apoptosis. **E** The effect of circMTO1 on cell migration. **P < 0.01, ^#^P < 0.05 compared with NC + ox-LDL + capecitabine. Ca. indicates the capecitabine

To further analyze the role of circMTO1 in ox-LDL-stimulated VSMCs, pcDNA 3.1-circMTO1 overexpression plasmid was transfected into VSMCs. As shown in Fig. 2, compared with the NC group, the expression of circMTO1 in the circMTO1 overexpression group was significantly increased (Fig. 2A, P < 0.01); ox-LDL stimulation significantly increased proliferation, but reduced apoptosis of VSMCs (Fig. 2B, P < 0.01). Interestingly, circMTO1 overexpression effectively attenuated ox-LDL-stimulated cell proliferation, promoted ox-LDL-stimulated cell apoptosis (Fig. 2D, P < 0.01), and inhibited ox-LDL-stimulated cell migration (Fig. 2E, P < 0.01). The above results indicated that circMTO1 overexpression inhibited the proliferation and migration of ox-LDL-stimulated VSMCs and induced apoptosis of ox-LDL-stimulated VSMCs.

### CircMTO1 served as a sponge of miR-182-5p

**Fig. 3:**
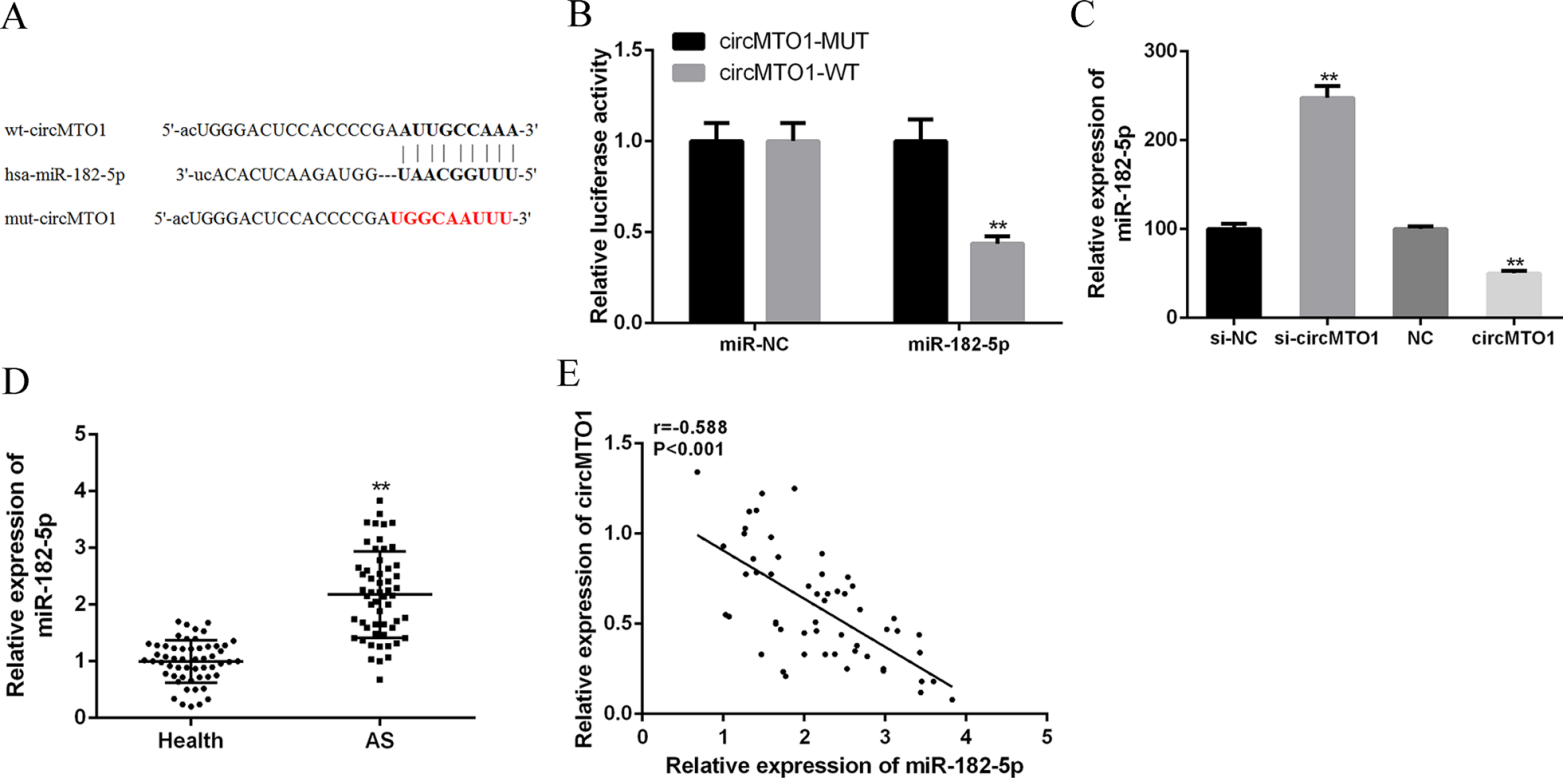
CircMTO1 acted as a sponge for miR-182-5p in AS. **A** Starbase predicted putative targeting sites for circMTO1 and miR-182-5p. **B** Luciferase activity in VSMCs co-transfected with miR-182-5p mimic and pmirg10-circMTO1-WT or pmirg10-circMTO1-Mut vector. **C** Effect of si-circMTO1 or circMTO1 overexpression plasmid on miR-182-5p expression in VSMCs. **D** Levels of miR-182-5p expression in serum of AS patients (n = 55) and healthy volunteers (n = 55). **E** Correlation of circMTO1 and miR-182-5p in serum of AS patients by Pearson analysis (n = 66). **P < 0.01, ***P < 0.001

Using the network tool Starbase, we predicted that circMTO1 might bind to miR-182-5p (Fig. 3A). As shown in Fig. 3B, we found that miR-182-5p overexpression significantly reduced the luciferase activity of wild-type 3′-UTR of circMTO1 (P < 0.01), but not the mutated 3′-UTR of circMTO1. Furthermore, as shown in Fig. 3C, compared with the NC group, circMTO1 knockdown significantly increased miR-182-5p expression in VSMCs, while circMTO1 overexpression significantly reduced miR-182-5p expression in VSMCs (P < 0.01). In addition, compared with the healthy group, serum miR-182-5p expression was significantly increased in AS patients (P < 0.01) (Fig. 3D), and there was a strong negative correlation (r = − 0.588, P < 0.001) between the expression of circMTO1 and miR-182-5p in AS patients (Fig. 3E). These results indicated that circMTO1 might exert its biological function through miR-182-5p sponging.

### CircMTO1 sponged and sequestered miR-182-5p to upregulate RASA1 expression

**Fig. 4:**
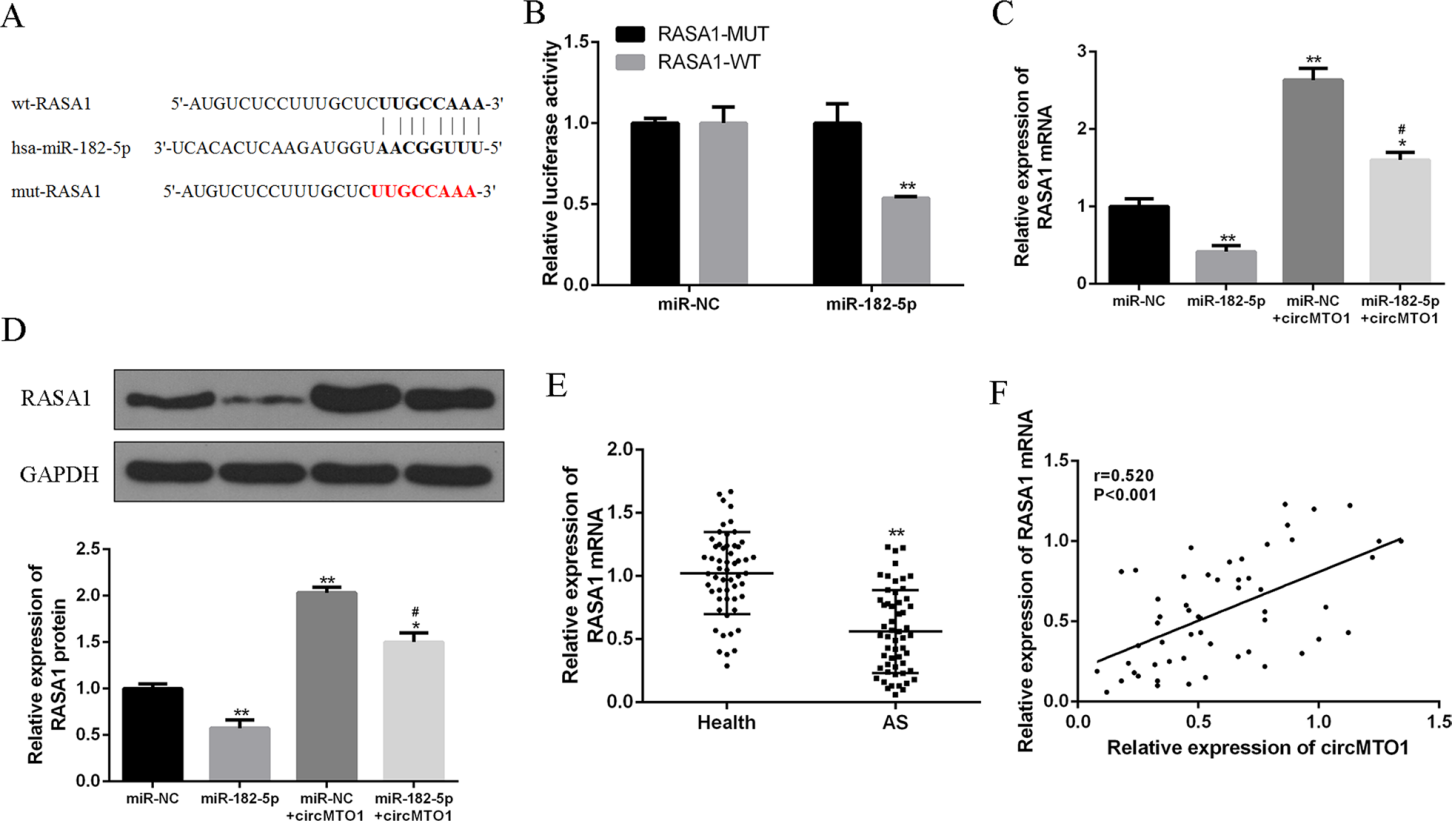
CircMTO1 upregulated RASA1 expression by sponging miR-182-5p. **A** Putative binding sites for miR-182-5p and RASA1 3′-UTR. **B** Luciferase activity in VSMC co-transfected with miR-182-5p mimic and pmirg10-RASA1 3′-UTR-WT or pmirg10-RASA1 3′-UTR-Mut vector. **C** RASA1 mRNA levels in VSMCs co-transfected with miR-182-5p mimic and circMTO1 vector. **D** RASA1 protein levels in VSMCs co-transfected with miR-182-5p mimic and circMTO1 vector. **E** RASA1 mRNA level in serum of AS patients (n = 55) and healthy volunteers (n = 55). **F** Pearson correlation analysis of circMTO1 and RASA1 in serum of AS patients (n = 55). * vs the control group, ^#^ vs miR-182-5p mock group. **P < 0.01, ^##^P < 0.01

Next, using Targetscan we predicted that RASA1 is a potential target for miR-182-5p (Fig. 4A). We found that miR-182-5p overexpression significantly reduced the luciferase activity of wild-type RASA1 vector (P < 0.01), but not mutated RASA1 vector (Fig. 4B). Furthermore, luciferase activity of the wild-type RASA1 vector was also decreased by co-transfection with pcDNA-circMTO1 (wt) but not with pcDNA-circMTO1 with the mutated miR-182-5p binding site (Fig. 4B). In addition, compared with the miR-NC group, miR-182-5p overexpression significantly reduced the mRNA and protein expression of RASA1 (P < 0.01), while circMTO1 overexpression significantly increased the mRNA and protein expression of RASA1 (P < 0.01). Co-transfection of circMTO1 with miR-182-5p reversed the effect of miR-182-5p overexpression on RASA1 expression (P < 0.05) (Fig. 4C and D). Besides, compared with the healthy group, RASA1 expression in AS patients was significantly reduced (P < 0.01) (Fig. 4E). Furthermore, there was a significant positive correlation between circMTO1 and RASA1 expression in AS patients (r = 0.520, P < 0.001) (Fig. 4F). These data showed that circMTO1 enhanced RASA1 expression by acting as a sponge for miR-182-5p in AS.

### CircMTO1 inhibited proliferation and migration of VSMCs by regulating the miR-182-5p/RASA axis

**Fig. 5:**
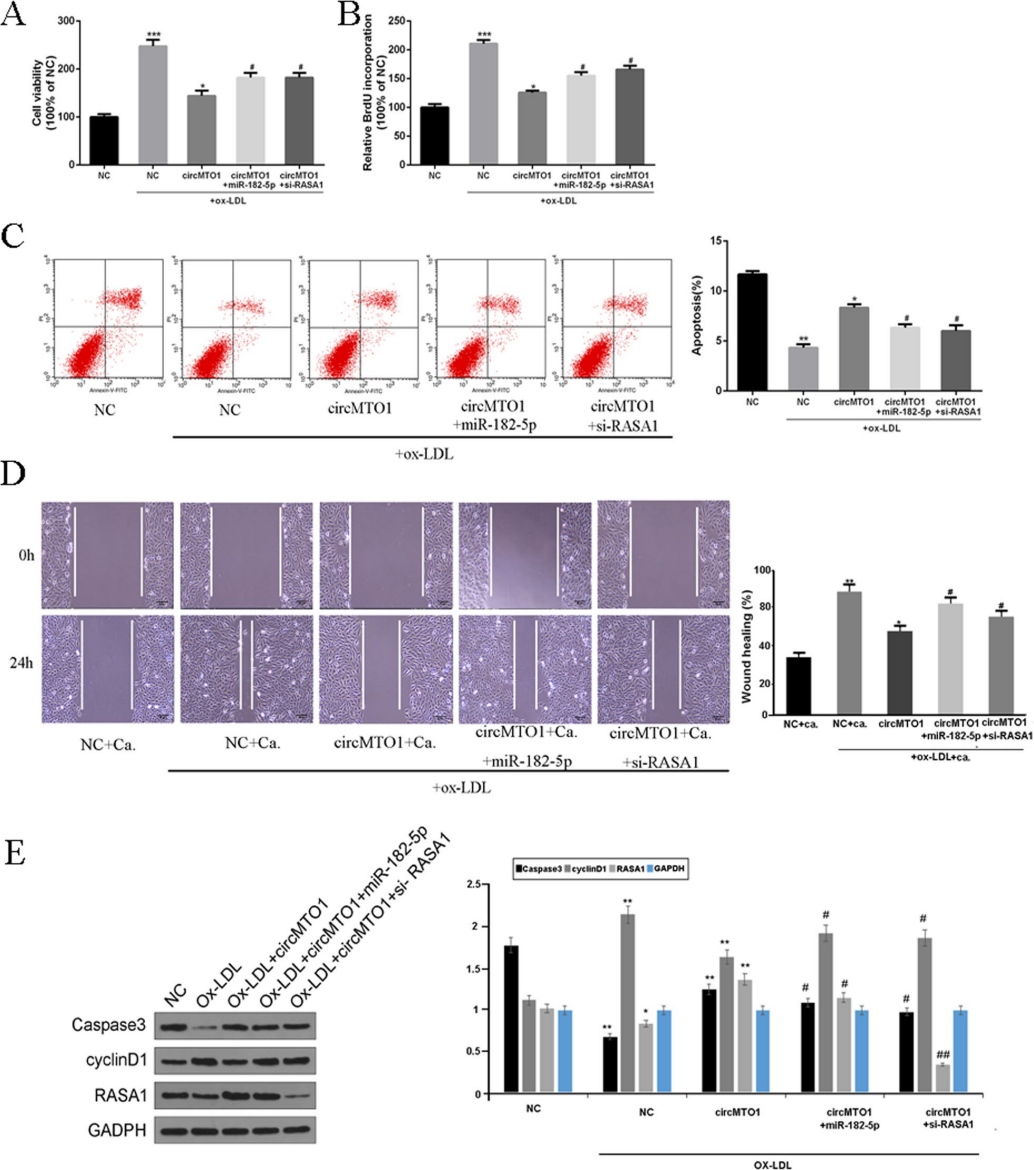
Effect of miR-182-5p overexpression or RASA1 knockdown on circMTO1-induced proliferation, apoptosis and migration of VSMCs. **A** Viability of VSMCs co-transfected with circMTO1 vector and miR-182-5p mimic or si-RASA1. **B** Proliferation of VSMCs co-transfected with circMTO1 vector and miR-182-5p mimic or si-RASA1. **C** Apoptosis of VSMC co-transfected with circMTO1 vector and miR-182-5p mimic or si-RASA1. **D** Migration of VSMCs co-transfected with circMTO1 vector and miR-182-5p mimic or si-RASA1. * vs NC (OX-LDL) + capecitabine group, ^#^ vs circMTO1(OX-LDL) + capecitabine group. Ca. indicates the capecitabine. ** P < 0.01, #P < 0.05. **E** Western Blot results of capase3, cyclinD1, RASA1 and GADPH in VSMCs co-transfected with circMTO1 vector and miR-182-5p mimic or si-RASA1. * vs NC group, ^#^ vs circMTO1 group. *** P < 0.001, ** P < 0.01, ^#^P < 0.05

To further confirm whether circMTO1 regulated AS progression via the miR-182-5p/RASA1 axis, the circMTO1 overexpression vector, miR-182-5p mimic, and siRNA-RASA1 were co-transfected into ox-LDL-stimulated VSMCs. As shown in Fig. 5A, B, compared with the NC group, circMTO1 overexpression significantly inhibited the viability and proliferation of VSMCs (P < 0.05), while co-transfection with miR-182-5p or si-RASA1 significantly abolished the proliferation inhibition by circMTO1 overexpression (P < 0.05). As shown in Fig. 5C and D, circMTO1 overexpression significantly induced VSMC apoptosis and inhibited VSMC migration, but these effects were abolished by co-transfection with miR-182-5p or si-RASA1 (P < 0.05). Moreover, some protein markers critical to apoptosis and proliferation were also regulated by the circMTO1/miR-182-5p-RASA1 axis (Fig. 5E). These results indicated that miR-182-5p overexpression or RASA1 knockdown reversed circMTO1-stimulated VSMC growth.

### circMTO1 overexpression attenuated arteriosclerosis by sponging miR-182-5p in vivo

**Fig. 6:**
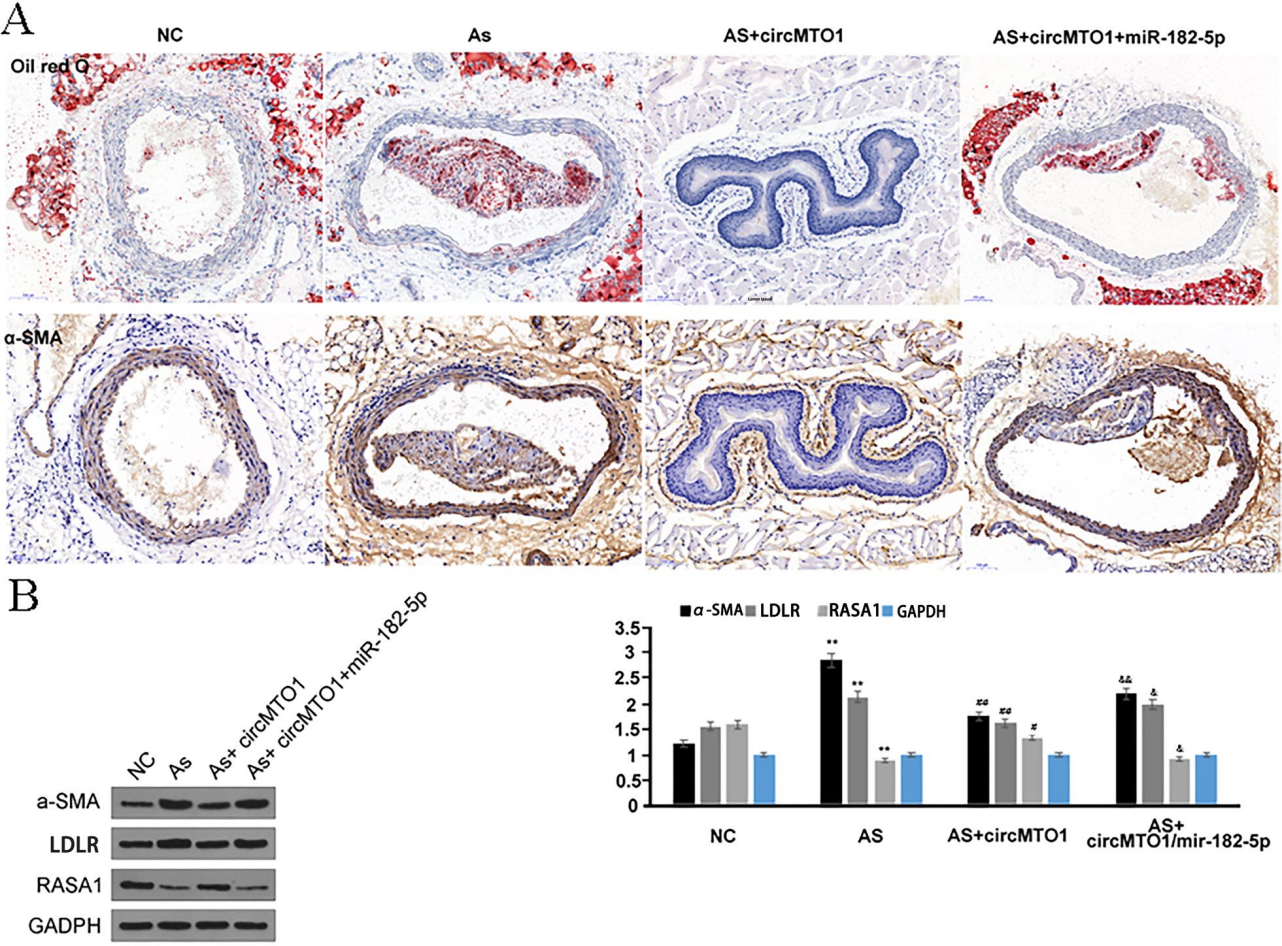
CircMTO1 overexpression attenuated arteriosclerosis by sponging miR-182-5p in vivo. **A** Representative microscopic images of the aortic root of a 36-week-old ApoE^−/−^ mice injected by AAV-modified vector via tail vein (AAV-PCDNA, AAV-pcDNA-circMTO1 and AAV-pcDNA-circMTO1/AAV-miR-182-5p). Lipid fixation was performed followed by oil red O (red) and smooth muscle α-actin (SMA, yellow) staining. **B** Western blot results of α-SMA, LDLR (LDL receptor), RASA1 and GADPH in the AS mice (AAV-PCDNA, AAV-pcDNA-circMTO1 and AAV-pcDNA-circMTO1/AAV-miR-182-5p). * vs NC group, ^#^ vs AS group, ^&^vs AS + circMTO1, **P < 0.01, *P < 0.05, ^##^P < 0.01, ^#^P < 0.05, ^&&^P < 0.01, ^&^P < 0.05

An AS mouse model was used to elucidate the function of the circMTO1/miR-182-5p axis, as described in the Materials and Method section, and AAV-pcDNA, AAV-pcDNA-circMTO1, and AAV-pcDNA-circMTO1/AAV-miR-182-5p were injected into these mice. As shown in Fig. 6A, compared with the control group, the histological evaluation of oil-red and the immunochemistry staining of α-SMA were both significantly increased in the AS group. circMTO1 overexpression significantly reduced the in situ staining of oil-red and α-SMA (Fig. 6A). Co-injection of AAV-circMTO1 with AAV-miR-182-5p partially rescued the in situ oil-red staining trend of α-SMA (Fig. 6A). RASA1 expression was also induced by injecting AAV-circMTO1 but attenuated by injecting AAV-miR-182-5p (Fig. 6B). The results indicated that circMTO1/miR-182-5p axis regulated the arteriosclerotic process.

## Discussion

Acute coronary events are common and severe diseases endangering human health (Wang et al. 2019b; Paigen et al. 1987). Various factors are involved in the pathogenesis and development of atherosclerosis. Among them, VSMCs play a critical role in intimal hyperplasia, plaque formation, and vascular stenosis (Arbabzadeh et al. 2012). VSMCs have high plasticity in terms of proliferation, migration, and transformation. When vascular injuries occur, VSMCs change from contractile type to synthetic type to promote blood vessels. However, under pathological conditions, the phenotype of VSMCs is dysregulated, which will lead to the development of cardiovascular diseases. Abnormal VSMC phenotype plays a critical role in atherosclerosis and other diseases after angioplasty (Zhao et al. 2014; Begum et al. 2011). Recently, it has been found that after vascular injury, some synthetic VSMCs are not derived from the transformation of contractile VSMCs but are derived from pluripotent vascular stem cells, suggesting that vascular wall-derived pluripotent stem cells differentiate into synthetic type VSMCs, which are important mechanisms for the development of atherosclerotic diseases (Mackay et al. 2011). The regulation mechanism in VSMC proliferation deserves further study.

With the deepening of basic research, circRNA has attracted more and more attention (Rubattu et al. 2014). In recent years, the critical regulatory role of circRNAs in cardiovascular diseases has gradually been recognized (Fan et al. 2017). For example, studies have found that circANRIL can bind to pescadillo homolog 1 (PES1), affecting pre-rRNA processing and ribosome production in VSMCs. CircANRIL can activate P53, induce apoptosis and inhibit proliferation(Nahapetyan et al. 2019). Studies have confirmed that circMTO1 plays a regulatory role in many diseases (Zhang et al. 2017). In glioblastoma, circMTO1 upregulation inhibits cell viability. In this study, we found that circMTO1 expression was decreased in AS patients and ox-LDL-stimulated VSMCs. Moreover, circMTO1 overexpression inhibited proliferation of ox-LDL-stimulated VSMCs. Therefore, circMTO1 can regulate VSMC proliferation and migration.

As a target of circRNAs, miRNAs are the most widely studied noncoding RNAs, regulating cell proliferation and participating in the process of development, body metabolism, and tumorigenesis (Song et al. 2017). Many miRNAs have been shown to affect VSMC migration through different target genes or different pathways (Han et al. 2017b). For example, miR-145 directly targets PDCD4 to promote VSMC proliferation (Amin and Lam 2015). Recent studies have shown that miR-155 inhibits VSMC migration and proliferation via endothelial nitric oxide synthase (Han et al. 2017b). MiR-182-5p is differentially expressed in various diseases (Xue et al. 2016; Zhang et al. 2015) and predicted as a target gene for circMTO1. The miR-182-5p expression level was upregulated in AS patients and in VSMCs by circMTO1 knockdown. There was a negative correlation between the expression of circMTO1 and miR-182-5p. Co-transfection of miR-125b-5p with circMTO1 reversed the effect of circMTO1 on VSMC proliferation. These data indicated that circMTO1 regulates VSMC growth by modulating miR-182-5p.

RAS p21 protein activator 1 (RASA1) is one of the 14 RAS GTPases involving cell differentiation and apoptosis (Guo et al. 2016b). It can regulate cell proliferation and migration under the action of various growth factors such as PDGF and CSF-1 (Yao et al. 2016). Compared with other members, the role of RASA1 in diseases is poorly understood. RASA1 has a tumor suppressor effect in colorectal and pancreatic cancers, but its role in VSMCs is still unknown (Chenghuan et al. 2013). In this study, we found that RASA1 is a potential target for miR-182-5p. RASA1 mRNA level was decreased in AS patients, and circMTO1 expression was positively correlated with RASA1 level in the serum of AS patients. In VSMCs, RASA1 expression was significantly decreased in the miR-182-5p overexpression group but significantly increased in the circMTO1 overexpression group. Co-transfection of miR-125b-5p with circMTO1 reversed the effect of miR-182-5p on RASA1 expression. In addition, co-transfection of si-RASA1 with circMTO1 reversed the effect of circMTO1 on VSMC proliferation. These indicated that circMTO1 inhibits proliferation and enhances apoptosis by regulating miR-182-5p/RASA1 in ox-LDL-stimulated VSMCs.

## Conclusion

CircMTO1 inhibits proliferation and migration and induces apoptosis by regulating the miR-182-5p/RASA1 axis in ox-LDL-stimulated VSMCs and might be a potential therapeutic target for AS.

## Supplementary Information

**Additional file 1:** The sequence of cirMTO1.

## Data Availability

The analyzed data sets generated during the study are available from the corresponding author on reasonable request.
