# Supplementary material for: CircMTO1 inhibits ox-LDL-stimulated vascular smooth muscle cell proliferation and migration via regulating the miR-182-5p/RASA1 axis
Source: Mol Med. 2021 Jul 8;27:73. doi: 10.1186/s10020-021-00330-2 (PMC8268171; doi:10.1186/s10020-021-00330-2)

**Additional data 1**

The sequence of cirMTO1

>hg19_hub_77_jeck_circRNAs range=chr6:74175932-74176329 5'pad=0 3'pad=0 strand=+ repeatMasking=none

GTCAGATGTCATGTAATCCTTCCTTTGGTGGCATCGGAAAGGGACATTTAATGAGGGAAGTAGATGCCTTGGATGGCCTGTGTTCTCGCATCTGTGACCAGTCTGGTGTACATTATAAAGTATTAAACCGGCGTAAGGGACCAGCTGTGTGGGGTCTGAGAGCTCAGATTGATAGGAAACTCTATAAACAGAACATGCAGGTAAGAATAGGGCATGAGCACAGGAAAGATTATAGTGATTGTTTAATTACCATGTTTCAACTGGCATTTTCTTTTGACAGAAAGAAATCTTGAATACACCACTGCTTACTGTTCAGGAGGGAGCTGTAGAAGATCTTATTCTTACAGAACCAGAGCCTGAACACACTGGGAAATGCCGTGTCAGTGGGGTTGTTTTGG

**The conservation analysis of cirMTO1 in 100 major vertebrates** (Data from UCSC: https://genome.mdc-berlin.de/cgi-bin/hgTracks?db=hg19&lastVirtModeType=default&lastVirtModeExtraState=&virtModeType=default&virtMode=0&nonVirtPosition=&position=chr6%3A74175931-74176329&hgsid=37799)


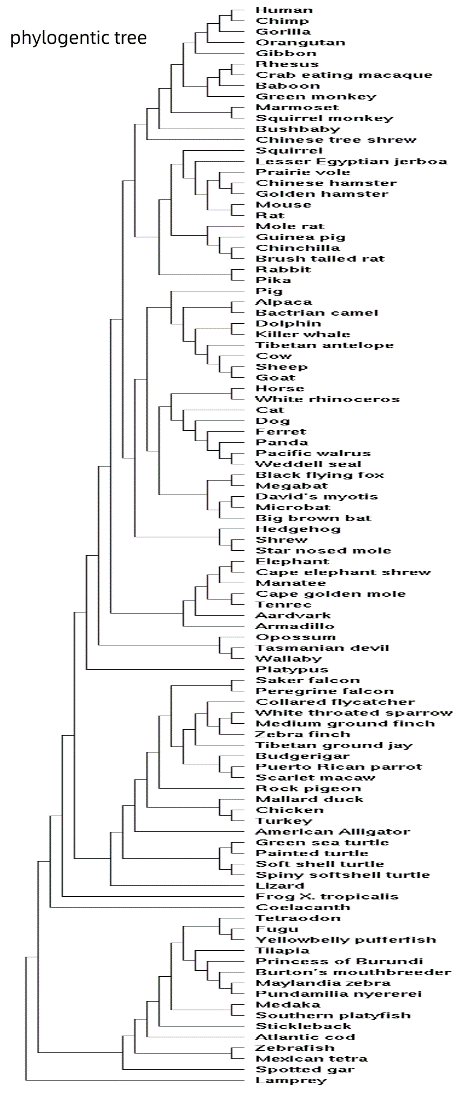

Supplement: Supplementary file 1 — Additional file 1: The sequence of cirMTO1. [file 10020_2021_330_MOESM1_ESM.docx]
